# Effect of Insurance Legislation on National Character in Germany

**Published:** 1913-10-25

**Authors:** 


### OUR NATIONAL INSURANCE ACT DEPARTMENT.

### LXXI.—Effect of Insurance Legislation on National Character in Germany.

A very remarkable article appeared in the *Post Magazine and Insurance Monitor* for October 11 under the title "The German Laws of Insurance." The author—Mr. Thomas Emley Young, a past-president of the Institute of Actuaries—was one of the earliest in this country to take an interest in the social legislation, and particularly the Insurance Laws, of Germany and other Continental countries. It was as long ago as April 1891 that he submitted a most exhaustive paper on the subject to the Institute of Actuaries, a paper which gave to Englishmen for the first time a concrete account of the proposals which were then receiving legislative sanction in Germany, and which was further illuminated by the inclusion of the statistical and mathematical foundations of the Law. At the time this paper was read—now more than twenty-two years ago—the author was a vice-president of the Institute of Actuaries; and he subsequently became president of that body. In his profession he has been considered an expert on all matters relating to social insurance legislation in Germany, and his remarks in the recent article already alluded to carry great weight.

Appearing in the *Post Magazine and Insurance Monitor* the article has doubtless been read by a wide circle of those who are interested in insurance in its practical aspects, but it is hardly likely that it will have that wider circulation which it merits. The widest publicity should be given to the facts cited by the author and to his opinions, for although the subject-matter is strictly confined to the effects of the German Insurance Laws, as indicated by the title, it is evident that a wider intention is implied, and that a warning is given against hasty legislation of a cognate character in our own country. It is with the hope of attracting public interest to the larger aspects of our own National Insurance legislation that we have considered it desirable to draw the attention of our readers to the weighty words of so high an authority on the effects which have resulted from similar legislation in Germany.

### The Sources of Information.

By way of introduction the author refers to his essay of April 1891, and recounts in the briefest form the prognostications of the anticipated inconsistency of the Act with the formation of individual development and national character. He then goes on to state that "the propitious time has now arrived, after virtually a generation of experience, for calm and unimpassioned survey and appreciation of the visible results. The evidence he adduces is not merely independent of official bias and trustworthy of nature, but forms also the practical and faithful observation and judgment of men of tested ability and character." The sources of his facts are three recent publications: (1) "The Undesirable Results of German Social Legislation," by Dr. Ludwig Bernhard, Professor of Political Science in the University of Berlin; (2) "The Future of Social Policy in Germany," by the same author; and (3) "The Practical Results of Working-Men's Insurance in Germany," by Dr. Ferdinand Friedensburg, late President of the Senate of the Imperial Insurance Office of Germany. In referring to the authors of these works Mr. Young remarks that they are "men of repute and trained capacity, and as the latter was engaged officially in the actual administration of the Act for twenty years, he speaks accordingly with the authority and insight of specific knowledge."

### Review of the Results of Social Legislation.

Then follows, under seven headings, the results of his observations, and we proceed to give extracts.

1. *The Limitation of Business Aptitude and Enterprise.*—The Act has distinctly weakened individual initiative and the sense of personal independence and responsibility, and has imposed a serious check upon commercial enterprise in competition with the markets of the world. Nearly every industry in Germany has preferred complaints of the State's disturbing intrusion into every phase of industrial life; the walls of workshops are literally plastered thickly with minute official rules and restrictions of work. Factories are continuously at the risk of the stoppage of work and enterprise from the official prescriptions . . . of hours of rest from labour. Government has intervened with arbitrary regulations of "rests," and thus the industry is endangered, the scope of its work is circumscribed, and its cost constantly increased; and to this must be added, as a consequence, the great rise in prices, to the detriment of personal well-being.

2. *The Propagation of Fraud.*—The Act, it is universally found, has proved a potent instrument to the organised perpetration of fraud. Accidentally, for example, it was discovered that a State pensioner was appearing as an acrobat; while another, who was receiving a pension for an injured elbow, defeated an opponent in a public pugilistic ring. Few convictions for pension frauds, it is true, have occurred; but this difficulty is explained by the fact that if every individual guilty of false allegations in order to secure a pension were placed upon trial, the numbers of the prosecuting attorneys and criminal judges would require to be doubled or trebled. . . . A class of "expert" assistants in fraud has sprung up since the origin of the Act, who travel from place to place, searching for prospective pensioners; stimulating (for a fee) all who may have suffered an injury to submit a claim however wild be the attempt, and even nefariously fabricating a claim where none existed. . . . The waiting-rooms of hospitals have been discovered to be additional centres of this corrupt tendency, where practised adepts in fraudulent demands teach their subterfuges and deceptive artifices to less enlightened sinners.

3. *Litigiousness.*—The enormous increase of litigation which has ensued can only be denominated a craze or mania; 170,000 out of 400,000 judgments have been appealed against, and so frivolous have been the subjects of these appeals that 80 to 90 per cent. had to be rejected. The extension of claims for trivial amounts is significant, as evidenced by the fact that from 1888 to 1908 the average amount per accident fell from 232 marks to 156 marks, although during the same period the total cost of indemnity rose from 5,900,000 marks to 155,100,000 marks.

4. *The Production of a Special Form of Disease.*—The existence of the Act and the right to a pension have exclusively directed the patient's mental attention hypochondriacally upon his physical condition in illness and injury, and thus have protracted or hindered a cure. The general nervous condition (actually and bodily intensifying every slight ailment) created by the connection between any illness and a title to pension has been described by physicians upon the Continent as "Pension-hysteria." The state may be alien from the intention of fraud, and forms a natural phenomenon which the law has unexpectedly involved. In commenting upon this particular result Mr. Young says: "It is obvious that, altogether apart from fraud, the extended prevalence of this condition, perfectly explicable on scientific principles, was an element which the Act (under whose influence it has originated) could never contemplate, and most materially disorganises the original provisions and estimates—thus rendering the augmented financial burd≀n of the Act an appalling tax upon the nation. But independently of this it is alarming to consider the decadent physical state induced in those who become its sufferers and (regarded from a national point of view) the deteriorated condition of body and mind which will be transmitted to the succeeding generation, with permanently baneful consequences upon both the physical vigour and the mental and moral character and energy of the community."

5. *Malingering and the Duration of Illnesses.*—The duration of the cure of ailments and accidents has been found to be greater than was the experience in pre-insurance days; for instance, the average period of invalidity connected with a broken collar-bone among insured persons in Germany has increased from forty days to eight months. . . . The State Fund is now viewed as an inexhaustible fount of charity. It is not to be wondered at that the official reports of the National Insurance Bureau in Germany now publicly enforce "in the urgent interests of the working classes as well as of the entire nation" the pressing duty of the administrators of justice to check the evil influences of covetousness for Insurance pay.

6. *Increasing Demands for Charity consequent upon the Act.*—Demands are constantly and imperatively made for electric treatment in the institutions which have been established; for massage; and for annual trips for the (so-called) restoration of health, but really for pleasurable holiday-making. . . . These demands become positive orders for the Government to obey. . . . The intended *help* is degenerating, as Dr. Friedensburg observes, into the cultivation of extended pauperism.

7. *Secondary Consequences of the Law.*—Each author dilates gravely upon what they term the "secondary" consequences upon social and national life which the Act has entailed. The authors have also pointed out, with especial emphasis, the undermining of the self-respect and energy of those whom it was the primary purpose of the Act to aid into more effective citizenship. The increasing demands for the extension of the benefits originally promised have resulted, by additional legislation, in deepening the social disaster, and have thus tended to degrade the authentic authority of the Government into a conscious or unconscious submission to the lower classes or the least righteously formative of the orders of the nation. As one of the authors sorrowfully deplores, the Act is definitely tending to induce new habits and a new conception of morality of an obnoxious type—habits which are alien from the customs of genuine citizenship; morality (or a code of conduct) which fails to be founded upon self-reliance, the righteous recognition of the claims of others, the educated sense of right and wrong, and the cultivation of manhood under high impulses and towards nobler ends. Even the elementary perception of rectitude is becoming obscured; and by certain classes the drawing of a pension, by whatever means obtained, is regarded as an honour: the ancient feeling of independence is vanishing. Dr. Friedensburg has expressed the conviction, from exceptional official observation, that the Insurance Law has thus unexpectedly conduced to a general demoralisation, and affirms that, in view of the experienced results, not a trace of the enthusiasm amid which the Law was inaugurated now survives, except in official reports. The other author has recorded the experience that manliness of endurance and the patient conflict with the ills of life are now found to lie outside the (so-called) working classes.

It should perhaps be pointed out that the Insurance Laws referred to cover invalidity insurance as well as sickness insurance, corresponding to our new National Insurance. That is to say, compensation for accidents and old-age pensions come within the scope of the Law. The criticism is, however, very striking, and whether we agree with the conclusions or not, it is clear that even the best devised schemes may fail through lack of insight into their secondary or unexpected results.

**NOTE.—Questions and Correspondence on all doubtful points are invited, and should be addressed to the Insurance Editor, The Hospital, 28 Southampton Street, Strand.**

